# Atypical Presentation of Systemic Arthritis in a Toddler with Down Syndrome

**DOI:** 10.1155/2021/6567770

**Published:** 2021-08-11

**Authors:** Emily Worley, Weijie Li, Jordan T. Jones

**Affiliations:** ^1^University of Kansas School of Medicine, Kansas City, KS, USA; ^2^Children's Mercy Kansas City, Kansas City, MO, USA; ^3^University of Missouri-Kansas City School of Medicine, Kansas City, MO, USA

## Abstract

Systemic juvenile idiopathic arthritis (sJIA) is a chronic, inflammatory disease of childhood, which is characterized by the combination of arthritis, serositis, daily, high-spiking fevers, and evanescent macular rash and can present with the life-threatening complication of macrophage activation syndrome (MAS). Children with Down syndrome (DS) have complex medical challenges related to abnormalities in their immune system, which can cause a broad spectrum of disease manifestations, which can occur atypically. Children with DS are at increased risk for arthritis and interstitial lung disease (ILD) associated with sJIA that has high mortality. This case report outlines an atypical presentation of sJIA in a 21-month-old male with DS in which fever was not part of the initial presentation of sJIA and then later developed MAS and ILD. Due to broad spectrum of disease and atypical presentation in children with DS, this case report was created to increase awareness of atypical presentations of rheumatic disease in children with DS.

## 1. Introduction

Down syndrome (DS) is one of the most common birth conditions in the United States of America, with approximately 5300 births annually, resulting in an estimated birth prevalence of 12.6 per 10,000 live births [1] and a population prevalence in the USA since 2010 of 6.7 per 10,000 inhabitants [2]. Children with DS have complex medical challenges related to abnormalities in their immune system that result in an increased rate of malignancy, infection, and autoimmune disease [3]. More recent reports suggest an increased incidence of arthritis in children with DS with the most presenting with polyarticular (five or more joints with active arthritis), antinuclear antibody (ANA), and rheumatoid factor negative disease (RF) [4]. Systemic juvenile idiopathic arthritis (sJIA) is a chronic, inflammatory disease of childhood that has an incidence of 0.4–0.9 per 100,000 in North America and Europe [5]. sJIA is characterized by the combination of arthritis, serositis, daily, high-spiking fevers, and evanescent macular rash and can present with the life-threatening complication of macrophage activation syndrome (MAS), which has a mortality rate of 8%–17% [6]. MAS is a form of secondary hemophagocytic lymphohistiocytosis (HLH) and manifests as a cytokine storm and presents with high, persistent fever, hepatosplenomegaly, cytopenias, transaminitis, and high serum ferritin. Therapies that antagonize cytokines interleukin- (IL-) 1 and IL-6 have been used to treat MAS effectively [6]. Reports suggest around two to three percent of children with DA will present with more sJIA-like arthritis [4, 7].

This report focuses on an atypical presentation of systemic arthritis and subsequent macrophage activation syndrome in a 21-month-old male with Down syndrome.

## 2. Case Presentation

A 21-month-old male with Down syndrome was admitted to the hospital for hypoxia, rhinitis, and nasal congestion due to viral bronchiolitis. He received supportive care and oxygen for the viral bronchiolitis, but during his admission was also noted to have rash and joint swelling. The rash had started about one month prior to admission and was noted to be intermittently present lasting days to hours prior to resolution. It was erythematous, macular, and did not appear pruritic or painful. There were no fevers during the previous month or with the current presentation. Joint swelling was noted in the knees, and the mother mentioned his knees would not straighten. He received home physical and occupational therapy, and they also noted inability to straighten his legs along with discomfort when they attempted to straighten his legs. The patient was also noted to have iron-deficiency anemia, poor weight gain, and neurotrophic keratitis as part of his Down syndrome. The mother was specifically concerned with his joints as she had a personal history of ankylosing spondylitis and maternal grandmother had rheumatoid arthritis; otherwise, there was no pertinent family history or regular daily medications. During hospital evaluation, laboratory tests were obtained and showed normal white blood cell count (WBC; 10.9 K/*μ*L), anemia (7.3 gm/dL), elevated platelet count (521 K/*μ*L), and appropriate complete metabolic panel including normal aspartate aminotransferase (AST) and alanine aminotransferase (ALT), with elevated c-reactive protein (CRP 5.8 mg/dL), erythrocyte sedimentation rate (ESR 63 mm/hr), lactate dehydrogenase (LDH 1109 units/L), ferritin (1971 nanograms/mL), fibrinogen (385 mg/dL), and positive HLA-B27. A peripheral smear was obtained and normal. A fecal calprotectin (286 mcg/gm; normal is ≤50) was elevated. A chest X-ray showed bilateral pleural effusions, an echocardiogram was completed and showed small pericardial effusion, and an ultrasound of the knees showed small joint effusions with mild synovial thickening. There was concern for inflammatory bowel disease with the poor weight gain, anemia, arthritis, and elevated fecal calprotectin, and CT enterography was performed, which showed a few segments of bowel wall thickening and groundglass opacities concerning for atelectasis. A colonoscopy was performed, and there was normal appearance of the gastrointestinal tract, and biopsies later revealed no abnormalities.

**Figure 1:**
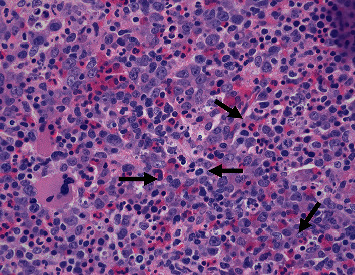
Bone marrow biopsy, H & E stain, hypercellular marrow, and increased histiocytes, which show hemophagocytosis (arrows).

**Figure 2:**
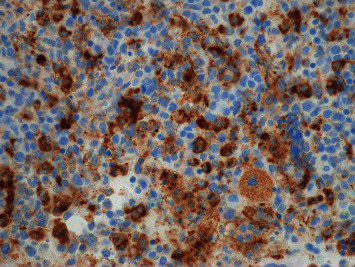
Bone marrow biopsy, CD68 immunohistochemistry stain, increased histiocytes, and hemophagocytosis.

The presentation was most consistent with sJIA, except there was no fever, which is a required criterion for sJIA diagnosis. There was also concern for MAS with the elevated ferritin and anemia; however, there was little additional supporting evidence for MAS. MAS is diagnosed in sJIA when there is fever, elevated ferritin (>684 ng/ml), and any two of the following: a platelet count of ≤181 K/*μ*L, AST > 48 units/L, triglycerides > 156 mg/dL, and fibrinogen ≤360 mg/dL [8]. Additionally, the diagnosis of sJIA should be excluded if there is ankylosing spondylitis in a first-degree relative [9]. Genetic testing for primary HLH was completed and did not identify any primary HLH, but did identify a heterozygous STXBP2 gene for a sequence variant designated c.190 C > T, which is predicted to result in the amino acid substitution p.Arg64Trp. This variant has not been reported in the literature; however, pathogenic variants have been reported in the STXBP2 gene, which cause primary HLH. The patient was started on naproxen, and there was noticeable improvement in his joint swelling and limitation after a few days. His supplemental oxygen was weaned off, and he was discharged when he no longer required support for his viral bronchiolitis. One month later, the patient was readmitted due to a two-day history of diarrhea and fever up to 103 degrees Fahrenheit. Upon admission, he was found to have elevated WBC (22.9 K/*μ*L), persistent anemia (7.0 gm/dL), normal platelet count (212 K/*μ*L), hyponatremia (132 mmol/L), elevated AST (237 unit/L) with normal ALT, elevated CRP (7.8 mg/dL), ESR (36 mm/hr), LDH (5621 units/L), ferritin (23697 nanograms/mL), and low fibrinogen (153 mg/dL). A respiratory viral panel was obtained and found to be negative, while chest X-ray showed pleural effusions. The results were most concerning for MAS. Infectious studies were conducted and found to be negative. A bone marrow biopsy was performed, which showed increased histiocytes with hemophagocytosis and was consistent with MAS (Figures 1 and 2). The patient was started on intravenous methylprednisolone (30 mg/kg) daily for five days and then transitioned to oral prednisolone (2 mg/kg divided twice daily) while simultaneously placed on anakinra, an IL-1 antagonist (8 mg/kg divided twice daily). The therapy resulted in significant improvement of inflammatory markers and clinical signs and symptoms. Prednisolone and anakinra were consolidated to once daily dosing and then weaned as an outpatient. Prednisolone was weaned and discontinued at six months, and anakinra was decreased to 5 mg/kg once daily dosing after isolated transaminitis occurred. After dose reduction, the transaminitis normalized, and he was continued on anakinra monotherapy. Later, the patient did have an sJIA flare with MAS that included arthritis, fatigue, and elevated inflammatory markers, cytopenia, and hepatosplenomegaly, and cyclosporine 1 mg/kg twice daily was added along with prednisolone 2 mg/kg/day. Shortly after his initial improvement, he developed erythematous clubbing of all his digits. Evaluation showed appropriate oxygen saturation level, and a chest CT scan was obtained, which showed interstitial lung disease. He was then switched from cyclosporine to tofacitinib (2.5 mg twice daily) and continued anakinra, and his clubbing has improved.

## 3. Discussion

Children with DS have complex medical challenges that present due to abnormalities in their immune system. This report highlights the complexity of disease that can present atypically in children with DS. The initial concerns for this patient were infection, malignancy, and inflammatory bowel disease due to the presentation of anemia, arthritis, and rash, especially in the setting of DS, as they have increased risk of malignancy and infection; however, they do not have any increased risk for inflammatory bowel disease [3]. Additionally, inflammatory bowel disease, specifically Crohn's disease, has been shown to be associated with juvenile idiopathic arthritis [10]; however, inflammatory bowel disease is rare in those with sJIA, and when it does occur, it is more commonly seen in older children [11]. Children with Down syndrome are at increased risk to develop Down syndrome-associated arthritis (DA), but it usually presents with polyarticular (five or more joints with active arthritis), antinuclear antibody (ANA), and rheumatoid factor negative disease (RF), rather than systemic disease involving hepatosplenomegaly, serositis, and lymphadenopathy [4]. Additionally, the patient has a mother with ankylosing spondylitis and is HLA-B27 positive, which is an exclusion criteria for systemic JIA [9]. It is possible that the previously unreported heterozygous STXBP2 gene variant is a pathogenic variant as pathogenic variants have been reported in the STXBP2 gene that cause primary HLH; however, there is an overlap between MAS in sJIA and primary HLH as 35% of patients with sJIA that presented with MAS had a heterozygous protein-altering rare variant in known gene that can cause primary HLH [12]. The arthritis, serositis, laboratory abnormalities, and classic rash mostly support the diagnosis of sJIA with MAS despite the patient not meeting criteria for sJIA due to the absence of quotidian-patterned, high-spiking fevers. The patient did meet criteria for MAS in sJIA and responded well to anakinra which has been shown to be an effective, beneficial therapy for MAS in patients with sJIA if used early in the disease course [13].

It should be noted that, in the earlier part of evaluation, this patient was noted to have groundglass opacities on CT scan as part of the evaluation for inflammatory bowel disease. It likely represents atelectasis per the official radiology read as the patient had a viral bronchiolitis at the time; however, the repeat CT scan that was completed when digital erythema and clubbing were noted showed subpleural reticulation and thickening consistent with interstitial lung disease and subpleural fibrosis. There are reports of an emergent high-fatality lung disease in patients with sJIA. The lung disease is associated with acute clubbing, digital erythema, eosinophilia, very elevated ferritin, MAS, cough, tachypnea, and hypoxia. The cause of the lung disease is unclear currently, but several theories have been raised such as adverse or delayed drug reaction to biologic therapy used to treat sJIA or possible underlying type-1-interferonopathy seen in those with Down syndrome. Regardless, there is increased risk for lung disease noted in children younger than five years of age with sJIA and those with Down syndrome [14]. Our patient has responded to the combination of anakinra and tofacitinib, which is a Janus Kinase (JaK) inhibitor. The addition of tofacitinib was made due to increased awareness that individuals with DS display hyperactivation of interferon signaling due to the extra copy of four interferon receptor genes encoded on chromosome 21 [15], which likely contributes to inflammatory disease in patients with DS, and JaK inhibitors decrease interferon activation.

Children with DS are at increased risk for autoimmune and autoinflammatory disease and frequently present with complex disease. Additionally, they can present atypically due to extensive variability in their immune system, making evaluation difficult. It is necessary for providers to have a broad differential when evaluating a child with DS.

## Data Availability

All data used to support the findings of this study are available upon request to the corresponding author.
